# Rapid Quantification and Validation of Biomarker Scopoletin in *Paederia foetida* by qNMR and UV–Vis for Herbal Preparation

**DOI:** 10.3390/molecules25215162

**Published:** 2020-11-06

**Authors:** Dai Chuan Tan, Alexandra Quek, Nur Kartinee Kassim, Intan Safinar Ismail, Joanna Jinling Lee

**Affiliations:** 1 Department of Chemistry, Faculty of Science, Universiti Putra Malaysia, UPM Serdang 43400, Selangor, Malaysia; tandaichuan@yahoo.com (D.C.T.); quek.alexandra@gmail.com (A.Q.); safinar@upm.edu.my (I.S.I.); 2 Integrated Chemical BioPhysics Research, Faculty of Science, Universiti Putra Malaysia, UPM Serdang 43400, Selangor, Malaysia; 3 Natural Medicines and Product Research Laboratory, Institute of Biosciences, Universiti Putra Malaysia, UPM Serdang 43400, Selangor, Malaysia; 4 Laboratory of Molecular Biomedicine, Institute of Bioscience, Universiti Putra Malaysia, UPM Serdang 43300, Selangor, Malaysia; joyousjoanna@gmail.com

**Keywords:** qNMR, scopoletin, *Paederia foetida*, method validation, quantification

## Abstract

Scopoletin has previously been reported as a biomarker for the standardization of *Paederia foetida* twigs. This study is the first report on the determination and quantification of scopoletin using quantitative nuclear magnetic resonance (qNMR) in the different extracts of *Paederia foetida* twigs. The validated qNMR method showed a good linearity (*r*^2^ = 0.9999), limit of detection (LOD) (0.009 mg/mL), and quantification (LOQ) (0.029 mg/mL), together with high stability (relative standard deviation (RSD) = 0.022%), high precision (RSD < 1%), and good recovery (94.08–108.45%). The quantification results of scopoletin concentration in chloroform extract using qNMR and microplate ultraviolet-visible (UV-vis) spectrophotometer was almost comparable. Therefore, the qNMR method is deemed accurate and reliable for quality control of *Paederia foetida* and other medicinal plants without extensive sample preparation.

## 1. Introduction

In the last decade, several studies have been conducted in an effort to investigate the effectiveness of medicinal plants for their use and consumption. Medicinal plants are regarded as the primary source of bioactive ingredients, particularly in the food and pharmaceutical industry, where they are used to add value to products [1,2]. Therefore, it is essential to identify compounds in plants that are known to have certain bioactive properties. The quality control systems applied in the herbal medicine industry are increasingly stringent. The recently defined quality control system for preparation of herbal products is based on several parameters, namely, authentication of the plant material and parts by classical taxonomy, phytochemical profiling (fingerprinting) of the herbal extracts through various chromatographic techniques, and quantitative assay of one or a few active phytoconstituents [3]. Thus, a universal and reliable analytical tool is required to obtain accurate and reliable results, especially for bioactive compound analysis of plant extracts.

*Paederia foetida* (Rubiaceae), locally called “Daun Sekentut”, is found in Asian countries, including Malaysia. Previous studies have shown that this plant exhibits good antidiabetic properties and have suggested dl-α-tocopherol, n-hexadecanoic acid, 2-hexyl-1-decanol, stigmastanol, 2-nonadecanone, cholest-8(14)-en-3-ol, 4,4-dimethyl-, (3β,5α)-, stigmast-4-en-3-one, stigmasterol, 1-ethyl-1-tetradecyloxy-1-silacyclohexane, ɣ-sitosterol, stigmast-7-en-3-ol, (3β,5α,24S)-, α-monostearin, and scopoletin as the bioactive compounds found in the chloroform extract [4,5].

**Figure 1:**
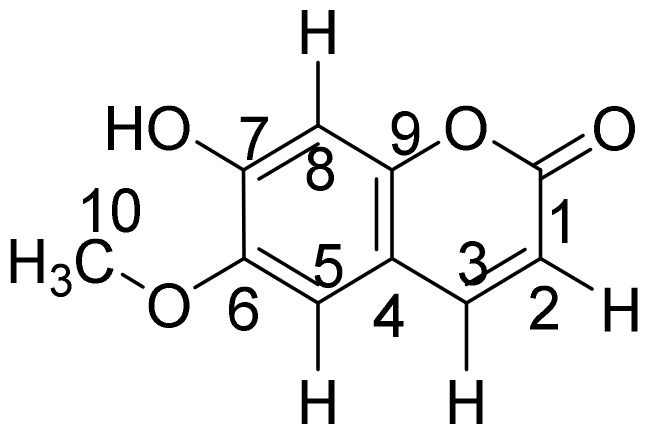
Structure of scopoletin.

Scopoletin (Figure 1) is a pharmacologically active coumarin compound found in various plant species. Previous studies have also shown scopoletin to exhibit fluorescence that is detectable in a fluorescence detector [6]. Compared to an ultraviolet-visible (UV-vis) detector, a fluorescence detector is more sensitive due to the improved signal-to-noise ratio and is more selective due to the usage of emission spectrum. The qualitative and quantitative analysis of scopoletin using high-performance liquid chromatography/ultraviolet (HPLC-UV) and HPLC-UV/fluorescence detector (HPLC-UV/FD) methods have also been previously reported. Furthermore, UV spectroscopy has also demonstrated desirable results toward the quantification of coumarin and phenolic compounds as these compounds can strongly absorb UV light while compounds that are naturally colored will lead toward an absorption in the visible range [7].

To date, there have been no reports on the quantification of scopoletin using quantitative nuclear magnetic resonance (qNMR), particularly in plant extracts. qNMR is a versatile approach that is widely used in the field of medicinal chemistry analysis. Compared to other spectroscopy methods, qNMR is a direct and straightforward assay with general applicability, limited method development time, and short analytical time. Most importantly, it is nondestructive [8]. Moreover, the content of compounds can be determined without any specific reference material. The application of qNMR has been developed to focus on its potential in the certification and quality control of reference compounds. Through this rapid and efficient process, the purity of natural products can be measured in a single analytical step without further supporting analysis while offering the option of retaining the substance [9]. Besides, qNMR holds the prospect of easy access owing to its strong characterization of natural products tested in vitro or in vivo for their biological and pharmacological effects. As such, the demand for compound certification is increasing.

Previous studies have reported less than 2.0% inaccuracy in qNMR analysis, which is an acceptable limit for precise and accurate quantification [10]. NMR has been proven to be a good analytical tool for quantitative estimation. In the present study, qNMR and UV-vis spectrophotometer were used to detect and quantify scopoletin as a bioactive compound in *Paederia foetida* chloroform extract. In addition, *Melicope latifolia* was also used to validate the method. These methods can be used for the quality control of *Paederia foetida* and other medicinal plants.

## 2. Results and Discussions

### 2.1. NMR Analysis

#### 2.1.1. Internal Standard (IS) Selection

In general, IS substances, such as trimethylsilylpropanoic acid (TSP), tetramethylsilane, and maleic acid, are widely used. The selection of a suitable IS depends on the compound of interest. The low boiling point and volatility of tetramethylsilane can lead to unfavorable results during quantitative analysis [11]. Maleic acid is not suitable as it is insoluble in deuterated chloroform (CDCl_3_), and the chemical shift of maleic acid is close to the signal of interest of scopoletin at 7.09 ppm. Considering the above deficiencies, TSP was selected as it has high purity. As such, the IS will not react with samples or solvents and will not produce an overlapping signal with samples. Its singlet peak at 0 ppm does not interfere with other possible peaks [11]. Therefore, TSP was chosen as the IS for calibration and quantitation in this study.

#### 2.1.2. Optimization of Solvents

For the NMR analysis, deuterated methanol (CD_3_OD) was selected for dilution as the chloroform extract cannot be dissolved in dimethyl sulfoxide-d_6_ (DMSO-d_6_) and TSP is not stable in CDCl_3_.

#### 2.1.3. Number of Scans

The number of scans is one of the main parameters that needs to be investigated to ensure that the signal-to-noise ratio is above 150 [12]. In this experiment, 8, 16, 32, 64, 128, and 256 scans were captured; eight scans were sufficient for the study.

#### 2.1.4. Pulse Angle and Relaxation Delay

The pulse angle and relaxation delay are used to achieve accurate quantification. The value of relaxation delay depends on the pulse angle used in the pulse program, and the longest T1 of all the signals is chosen for quantification in experiments [12]. A 90° pulse angle was used in the present study. The relaxation delay should be long enough to reach a quantitative condition observable on the stability of the integral area. To do so, the ^1^H-NMR spectra was accumulated using different relaxation delays, with scopoletin of a known purity and the IS representing the sample. Relaxation delays of 2, 5, 20, 40, 60, and 120 s were conducted on the integral area. The integral area showed almost no difference and no influence on the quantitative data [13]. Therefore, 60 s was chosen as the relaxation delay.

#### 2.1.5. ^1^H-NMR Spectra of Extracts

**Figure 2:**
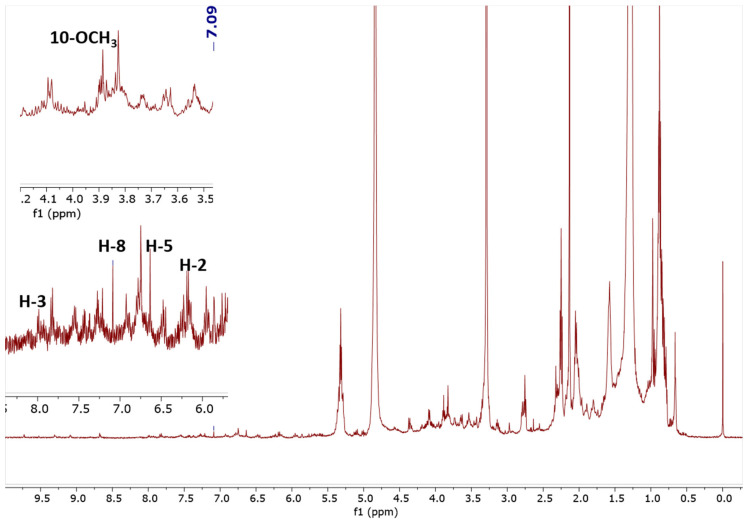
^1^H spectrum of *Paederia foetida* twigs chloroform extract.

**Figure 3:**
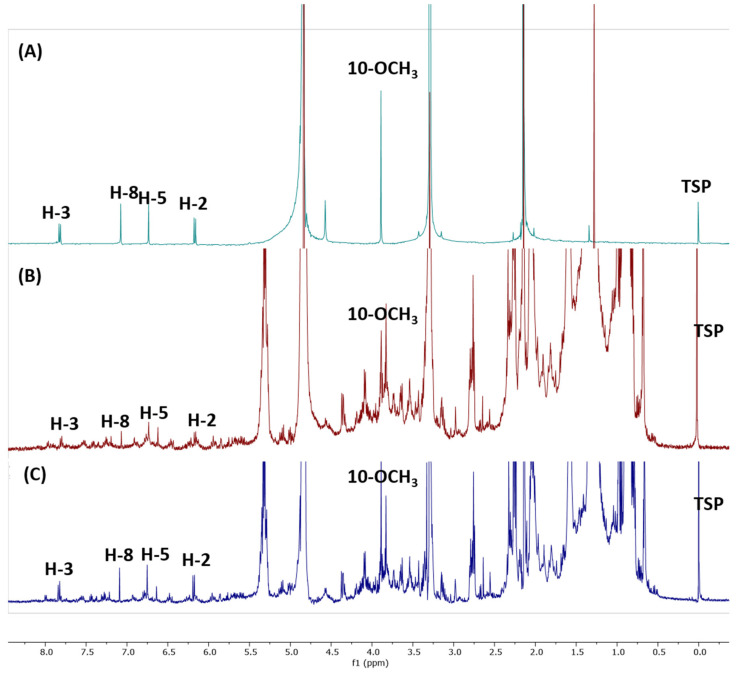
Stacked spectra of scopoletin standard (**A**), *Paederia foetida* twig chloroform extract before recovery analysis (**B**), and *Paederia foetida* twig chloroform extract after recovery analysis (**C**).

**Figure 4:**
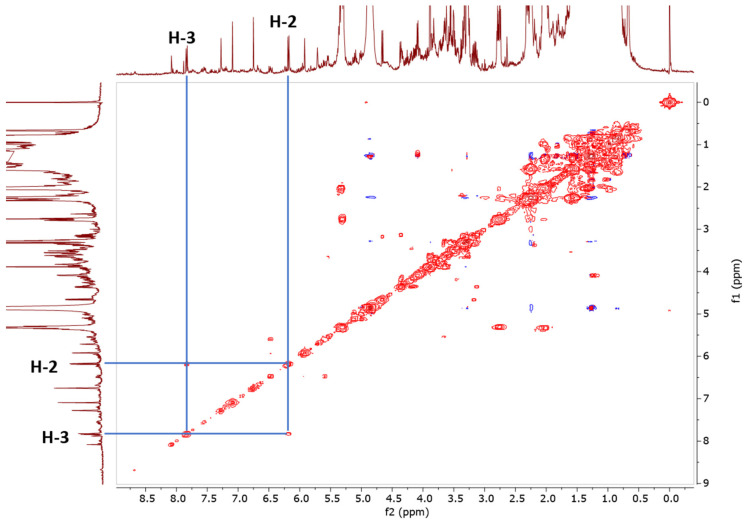
A correlation spectroscopy (COSY) spectrum of *Paederia foetida* twig chloroform extract.

The ^1^H-NMR spectra were recorded on a JEOL ECX 500 NMR spectrometer operating at 500.16 MHz equipped with a 5 mm inverse probe. Figure 2 illustrates the ^1^H-NMR spectrum of the chloroform extract. The spectra indicate signals of CD_3_OD (δ 3.35 ppm; δ 4.87 ppm), the internal standard scopoletin (δ 7.09 ppm), and TSP as the reference standard, calibrated to δ 0.00 ppm. The singlet signal at δ 7.09 ppm (H-8) belonging to the CH of scopoletin was used for the quantification. The stacked spectra of the scopoletin standard and chloroform extract are shown in Figure 3. Due to the signal overlap in the ^1^H-NMR spectra, two-dimensional NMR, i.e., proton-proton correlation spectroscopy (COSY), was used to overcome these limitations. The presence of scopoletin in the plant extracts was confirmed using COSY (Figure 4). The COSY spectrum clearly showed a cross-peak correlation of H-2 and H-3; therefore, the correlation confirmed the presence of scopoletin in the extracts [14].

#### 2.1.6. Specificity and Selectivity

Specificity and selectivity are key prerequisites that must be evaluated in order to avoid possible interference according to the presence of metabolites in plant extracts or solvents [15]. Figure 3 shows the ^1^H-NMR spectra of the IS and plant extracts. It is obvious that the signal obtained at 7.09 ppm for scopoletin was not disturbed by the solvent (3.35 and 4.87 ppm).

#### 2.1.7. Linearity

Linearity tests were performed using the standard solution of scopoletin at different concentrations ranging from 0.012 to 1.50 mg/mL. The linearity regression yielded a correlation coefficient of 0.9999 and a regression equation of *y* = 1.0325x − 0.0057. The correlation coefficient was close to 1.0000, indicating good linearity.

#### 2.1.8. Accuracy

The accuracy of qNMR was evaluated by the recovery test, in which a known quantity of scopoletin (at 80%, 100%, and 120%) was added into the chloroform extract after solvent extraction. The accuracy was calculated with Equation (1), and the average recoveries were recorded at 94.08%, 98.93%, and 108.45% with the relative standard deviations (RSDs) of 1.06%, 1.00%, and 0.39%, respectively. According to the Association of Official Analytical Chemist (AOAC) and Codex Alimentarius Commission (CODEX) [16,17], the acceptable range of recovery is between 90% and 107%. However, about 95% of the resulting mean recoveries for a typical performance in a single laboratory falls within the range of 80–120% [16]. Even so, some laboratories may accept method performance criteria that falls outside of the required criteria when very low concentrations are applied [17]. Furthermore, in the United States Food and Drug Administration (USFDA) guidelines for method validation, the acceptability criterion for recovery is between 80% and 110% [18]. Thus, the method was validated as the three different concentrations were found within the specified range. The values of %recovery and %RSD indicated that the method was accurate. Table 1 suggests that the qNMR method is able to provide superior accuracy for scopoletin determination.

Recovery (%) = (A_x_ − A_o_)/A_s_ × 100% (1)

where A_x_ is the calculated amount of analyte after reference addition, A_o_ is the calculated amount of original analyte before reference addition, and A_s_ is the true amount of reference addition.

#### 2.1.9. Precision

The precision was determined by repeatability and intra- and interday precision. The repeatability (Table 2) was tested six times, while the intra- and interday precision (Table 3) were tested with different time intervals and days, respectively. The %RSD of the repeatability and intra- and interday precision were 0.23961%, 0.02468%, and 0.03702%, respectively. The RSD of the analysis was lower than 2%, which denotes acceptable repeatability and precision.

#### 2.1.10. Limit of Detection and Quantification

The limit of detection (LOD) and limit of quantification (LOQ) were determined based on the standard response deviation and the slope of the regression line to perform the sensitivity analysis. LOD and LOQ were calculated based on the signal-to-noise ratio with LOD = 3 × S/N and LOQ = 10 × S/N [19]. The LOD showed the lowest amount of standard in a sample of 0.009 mg/mL, which can be detected but not necessarily quantified as an exact value [20]. The LOQ showed the lowest amount of standard in a sample with a quantitation limit of 0.029 mg/mL, which can be quantitatively determined with suitable precision and accuracy [20].

#### 2.1.11. Sample Stability

Stability analysis is essential to evaluate if there is significant variation after the initial solution is stored for a period of time [21]. The stability of scopoletin solution was measured by comparing the amount of scopoletin present in the initial sample versus the sample stored for 0, 8, 16, 24, and 48 h at room temperature, as shown in Table 4. The RSD was 0.022%, indicating that the scopoletin solution was sufficiently stable during the analysis period.

#### 2.1.12. Robustness

The robustness of the method was evaluated by varying four parameters, namely, the number of scans, relaxation delay, acquisition time, and spectral width. All the samples were freshly prepared for each parameter adjustment. The scopoletin amount measured with optimal parameters was 100.08%. The robustness analysis is tabulated in Table 5. The maximum difference of 0.43% showed that the parameters did not significantly alter the results compared with the optimized state [21].

### 2.2. UV-Vis Spectrophotometer Analysis

#### 2.2.1. Optimization of Solvents

The polarity of the solvent affects the quality and sensitivity of the spectrophotometer result. Scopoletin is slightly soluble in distilled water but very soluble in methanol. Standard scopoletin and plant extracts were first dissolved in methanol during the development and optimization phase and diluted to the desired concentration using distilled water. The maximum absorption of standard scopoletin was at a wavelength of 344 nm.

#### 2.2.2. Linearity

**Figure 5:**
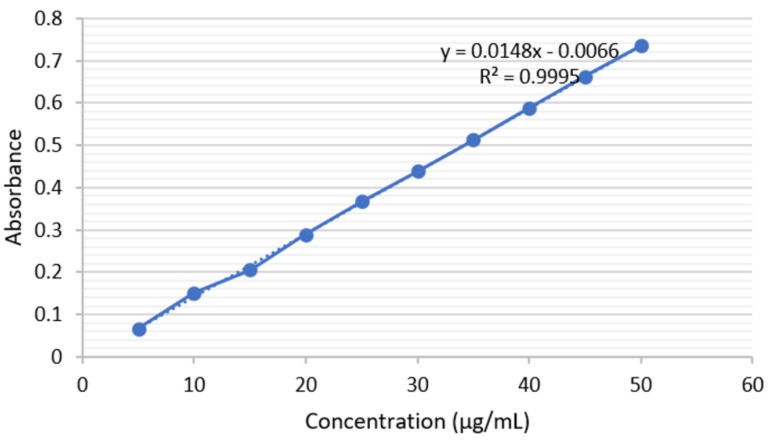
Calibration curve of standard scopoletin at 344 nm.

A concentration range of 5–50 µg/mL of standard scopoletin was used in the linearity study and determined at a wavelength of 344 nm. The regression equation was recorded to be *y* = 0.0148x − 0.0066 with a correlation coefficient of 0.9995, as shown in Figure 5. Thus, a good linear correlation was observed between the absorbance and the concentration of standard scopoletin.

#### 2.2.3. Limit of Detection and Limit of Quantification

The LOD and LOQ for the UV-vis spectrophotometer were 2.238 and 7.463 µg/mL, respectively. In this study, 20 µg/mL concentration of standard scopoletin was used for the validation.

#### 2.2.4. Accuracy

The percentage recovery of standard scopoletin was determined by the developed method at 80%, 100%, and 120% sample concentration ranging from 100.25% to 105.37% (Table 6). The range of the results was within the acceptability range according to the AOAC and CODEX. The values of %recovery and %RSD indicated that the method is accurate.

#### 2.2.5. Precision

The precision of the method was studied on the repeatability and intra- and interday precisions. The intra- and interday precisions were determined using three different concentrations with triplicates. The repeatability study was carried out with six replicates at the same concentration. Table 7 showed a low %RSD with the value of 0.569. Based on the results from Table 8 and Table 9, all the %RSDs were found to be within the limit of 2.0%. This indicates that the developed and validated method is of high precision. Besides that, %RSD with low values also showed the repeatability of the method. Therefore, the precision results showed significant reproducibility [22]. The detected absorbance is affected by various factors, such as sample concentration, temperature, factors inherent to the procedure, specific circumstance occurring on a particular day, the analyst, the instrumentation, the laboratory, etc. [23]. A trend of decrease in absorbance with increase in temperature has previously been observed [24,25].

#### 2.2.6. Robustness and Ruggedness

Robustness and ruggedness are the ability of the developed method to remain unaffected by small variations in parameters and environmental conditions during normal usage. The robustness was conducted by varying the wavelengths. As can be seen in Table 10, the %RSD of 339, 344, and 349 nm were 0.608%, 0554%, and 0.557%, respectively, which is not more 2.0%. The developed method is highly reliable and stable with small values of %RSD. To test the ruggedness of the method, two analysts (Dai Chuan Tan and Alexandra Quek) conducted the same procedure under the same conditions. Table 11 demonstrates the stability of the developed method with %RSD of 1.038 and 1.033, respectively.

### 2.3. Application to Herbal Medicine Using qNMR and UV-Vis Spectrophotometer

In terms of repeatability, linearity, accuracy, and precision, both the qNMR and UV-vis spectrophotometer techniques demonstrated excellent performance for the quantification of scopoletin in medicinal plant extracts. A comparison of *P. foetida* and *Melicope latifolia* extracts using these two techniques reflected good results, as tabulated in Table 12. The *P. foetida* chloroform extract showed 7.34% scopoletin content, while the other two solvent extracts (hexane and methanol) did not show any scopoletin content due to the absence of the peak in ^1^H-NMR. Scopoletin was only detected in the semipolar solvent (chloroform), which in in agreement with a previous study [5]. Differences were observed between the amount of scopoletin in *P. foetida* chloroform extract as determined by the qNMR and UV-vis spectrophotometer. qNMR showed higher scopoletin content compared to the UV-vis spectrophotometer. The results indicate that the qNMR method is as effective as the UV-vis spectrophotometer despite differences in the basic principle of the techniques. The UV-vis spectrophotometer is based on the principle of light absorption, thus requiring a previous matrix effect evaluation in order to use the calibration curve approach, whereas the qNMR responds uniformly to hydrogen atoms [26,27]. In addition, the number of ^1^H-NMR signal corresponds directly to the number of nuclei responsible for that signal. Besides that, *M. latifolia* also showed high scopoletin content using the qNMR method, which suggests that the quantification method can be similarly applied on other medicinal plants.

Although both techniques fulfilled the method validation requirements, qNMR provided better results due to its higher precision and repeatability. While the preparation of both techniques are simple and fast, qNMR has several advantages, including excellent reproducibility, automation, quantification without identical standard material, and total detection, permitting an unbiased overview of the sample composition [26]. Some difficulties may appear in UV-vis, causing inefficient drug quantification to occur. This could be due to the possible interactions among components, especially when the maximum absorbance of the drug is close to the maximum absorbance of an eventual component of the formulation [26]. Thus, qNMR can be a more efficient quality control tool for the herbal industry to regulate the formulation of herbal medicines.

## 3. Materials and Methods

### 3.1. Chemical and Reagents

Methanol-d_4_ (CD_3_OD, 99%), deuterated chloroform (CDCl_3_), dimethyl sulfoxide-d_6_ (DMSO-d_6_), 3-(trimethylsilyl)propionic-2,2,3,3-d_4_ acid sodium salt (TSP-d_4_, 98% deuterated), and analytical-grade solvents were purchased from Sigma (Merck KGaA, Darmstadt, Germany). The scopoletin standard (99%) was purchased from Sigma (Sigma-Aldrich, St. Louis, MO, USA).

### 3.2. Sample Collection and Extraction

*Paederia foetida* was collected from Johor, Malaysia, on 7 June 2017. A certified botanist authenticated the plant at the Institute of Bioscience, Universiti Putra Malaysia (UPM), whereby the specimen voucher (SK3177/17) was deposited. The plant twigs separated from the leaves were dried at room temperature for two weeks and ground into powder [5]. The powdered plant twigs (900 g) were macerated in hexane solvent over 72 h, filtered, and its residue was re-extracted twice in the same manner with a fresh batch of solvent. The collected filtrates were pooled and concentrated using a rotary evaporator at 40 °C. The obtained crude hexane extract was stored at 4 °C for further use. Similarly, the above extraction method was repeated thrice using chloroform, followed by methanol. In addition, another medicinal plant was also collected for the quantification of scopoletin. *Melicope latifolia* (DC.) T. G. Hartley was collected from Bukit Serting, Negeri Sembilan, and certified in the Department of Chemistry, Universiti of Malaya, with the accession number KL5538.

### 3.3. Quantitative Nuclear Magnetic Resonance

#### 3.3.1. Preparation of Standard and Internal Standard Solutions

Scopoletin standard was accurately weighed and dissolved in CD_3_OD to obtain a working solution at a final concentration of 1.5 mg/mL. The IS stock solution was prepared by dissolving TSP-d_4_ in CD_3_OD, which was then diluted with CD_3_OD to obtain a final concentration of 0.01% (0.1 mg/mL).

#### 3.3.2. Sample Preparation

The plant extracts (30.0 mg) were weighed in a 1.5 mL Eppendorf tube and dissolved with CD_3_OD to the volume of 700 µL. The extract solution was then sonicated for 15 min at 25 °C and centrifuged at 13,000 rpm for 10 min. Subsequently, 350 µL of the extract solution was transferred into another 1.5 mL Eppendorf tube and diluted with 350 µL IS stock solution. Finally, 600 µL of each mixture was transferred to a 5 mm NMR tube.

#### 3.3.3. ^1^H-NMR Acquisition Parameter

The qNMR analysis was carried out using a 500 MHz irradiation frequency, direct liquid probe temperature of 25 °C, 8 scans, 15 ppm spectral width, 4 s acquisition times, 60 s relaxation delay, and 90° pulse angle. All the data processing was performed using the Mestre Nova software. During data processing, the phase and baseline were corrected manually, and the signals were also integrated manually. The chemical shift of all data was referenced to the TSP-d_4_ peak at 0 ppm.

#### 3.3.4. qNMR Analysis

The signal response (integrated signal area/intensity) in the ^1^H-NMR spectrum (*I_x_*) is directly proportional to the number of nuclei (*N_x_*) generating the corresponding resonance line, as shown in Equation (2):

*I_x_* = *K_s_N_x_* (2)

*K_s_* is an unknown spectrometer constant, whereas it is constant for all resonance lines in the same ^1^H-NMR spectrum. Accordingly, the determination of relative area ratios (*I_x_/I_y_*) is the most efficient way to obtain quantitative results using the equation below:

(3) $$\frac{I_{x}}{I_{y}}=\frac{N_{x}}{N_{y}}$$

The following formula calculates the concentration of the compound:

(4) $$C_{x}=\;\frac{I_{x}}{I_{std}}\;\times \;\frac{N_{std}}{N_{x}}\;\times \;\frac{M_{x}}{M_{std}}\;\times \;\frac{P_{std}}{P_{x}}\;\times \;\frac{V_{std}}{V_{x}}\;\times \;C_{std}$$

where *C_x_* is the analyte concentration (compound), *C_std_* is the concentration of internal standard solution, *I_x_* is the integral value of the ^1^H signal of the analyte, I_std_ is the integral value of the ^1^H signal of the IS, *N_x_* is the number of protons for the analyte, *N_std_* is the number of protons for the IS, *M_x_* is the molar mass of the analyte, *M_std_* is the molar mass of the IS, *P_x_* is the purity of the analyte, *P_std_* is the purity of IS, *V_x_* is the volume of sample, and *V_std_* is the volume of IS.

#### 3.3.5. COSY Measurement Parameter

COSY analysis was carried out using a 500 MHz irradiation frequency, probe temperature of 25 °C, 24 scans, 15 ppm spectral width in both dimensions, 0.14 s acquisition time, and 1.5 s relaxation delay. The number of collected data points was 1280, and the number of time increments was 256. The squared sine apodization function was applied in both dimensions prior to Fourier transformation [28]. The data matrix after the Fourier transformation was 2048 × 2048.

#### 3.3.6. Method Validation

The analytical validation of the method was done according to the International Conference on Harmonization (ICH) guidelines Q2(R1) [20]. The following parameters were evaluated in qNMR analysis: linearity and range, accuracy, precision, stability, and robustness. The mean, standard deviation, and RSD were calculated.

### 3.4. UV-Vis Spectrophotometer

#### 3.4.1. Preparation of Standard and Internal Standard Solutions

Here, 500 µg/mL of scopoletin standard stock solution was prepared and diluted with methanol. The 5–50 µg/mL concentration of scopoletin was prepared to form the stock solution with distilled water as diluent. The wavelength of scopoletin was scanned. The maximum absorption of scopoletin was 344 nm, which is similar to the study by Ferdinal et al. [29].

#### 3.4.2. Sample Preparation

For the UV-vis spectrophotometer analysis, plant extracts were prepared in a 50 mL volumetric flask using methanol to give a final concentration of 500 µg/mL. The solution was then diluted to a concentration range of 20–50 µg/mL using distilled water as a diluent.

#### 3.4.3. Microplate UV-Vis Spectrophotometer Conditions

The Bio-Tek µQuantTM single-channel microplate spectrophotometer was used in the analysis. The instrument has a long-life xenon flash source and a monochromator with a wavelength range of 200–999 nm.

#### 3.4.4. Method Validation

The analytical validation of the method was done according to the International Conference on Harmonization (ICH) guidelines Q2(R1) [20]. The following parameters were evaluated using UV-vis analysis: linearity and range, accuracy, precision, repeatability, robustness, and ruggedness. The mean, standard deviation, and RSD were calculated.

## 4. Conclusions

A fast and reliable qNMR method was developed and used to measure scopoletin content in *Paederia foetida* chloroform extract. The content determination results using qNMR were compared continuously with UV-vis spectrophotometer. The qNMR method is more straightforward in its sample preparation, faster in analysis, less rigorous in experimental conditions, and has better repeatability than the UV-vis spectrophotometer. qNMR is a specific, accurate, precise, simple, and repeatable method for scopoletin determination in *P. foetida* extracts. It can also achieve precise quantification without any analyte reference material. Thus, the established qNMR method is an excellent option for routine quality control and stability analysis of scopoletin in herbal medicines. This method also introduces various possibilities for the quantitative determination of biomarkers, especially for compounds with low yield.

## Figures and Tables

**Table 1:** Accuracy test results using quantitative nuclear magnetic resonance (qNMR).

Accuracy Level (%)	Scopoletin in Extract (A_o_)	Scopoletin Added (A_s_)	Scopoletin Obtained (A_x_)	Recovery (%)	Analysis
80	0.03130	0.02500	0.05510	95.20	Mean = 94.08 %RSD = 1.06
	0.03200	0.02560	0.05600	93.75	
	0.03160	0.02530	0.05520	93.28	
100	0.02250	0.02700	0.05170	108.15	Mean = 108.45 %RSD = 0.39
	0.02260	0.02350	0.04820	108.94	
	0.02240	0.02180	0.04600	108.26	
120	0.04000	0.06000	0.10000	100	Mean = 98.93 %RSD = 1.00
	0.04320	0.06120	0.10320	98.04	
	0.03950	0.04800	0.08690	98.75	

Note: RSD means relative standard deviation. The amount unit is mg/mL.

**Table 2:** Determination of repeatability by six replicate and analysis using qNMR.

No.	Calculated Concentration (mg/mL)
1	0.03130
2	0.03140
3	0.03150
4	0.03140
5	0.03150
6	0.03140
Mean	0.03142
Standard deviation	0.00007
%RSD	0.23961

**Table 3:** Precision tests of the scopoletin amount using qNMR.

No.	Intraday Calculated Concentration (mg/mL)			Interday Calculated Concentration (mg/mL)		
	9.00 a.m.	11.00 a.m.	1.00 p.m.	Day 1	Day 2	Day 3
1	0.04427	0.04425	0.04424	0.04427	0.04424	0.04422
2	0.04426	0.04425	0.04425	0.04426	0.04425	0.04423
3	0.04427	0.04426	0.04426	0.04427	0.04425	0.04423
4	0.04427	0.04427	0.04427	0.04427	0.04426	0.04424
5	0.04425	0.04424	0.04424	0.04425	0.04425	0.04423
6	0.04426	0.04425	0.04425	0.04426	0.04425	0.04422
Mean	0.04426			0.04425		
Standard deviation	0.00001			0.00002		
%RSD	0.02468			0.03702		

**Table 4:** Stability results of standard scopoletin using qNMR.

Time (h)	Assay (%)	Differences (%)
0	99.70	NA
8	99.70	0.00
16	99.70	0.00
24	99.69	0.01
48	99.65	0.04
Average	99.69	
%RSD	0.022	

**Table 5:** Robustness analysis of scopoletin using qNMR.

Parameters (Target Value)	Change	Assay (%)	Differences (%)
Number of scans (8)	4	99.65	0.43
	12	100.02	0.06
Relaxation delay (60 s)	50 s	100.05	0.03
	70 s	99.98	0.10
Acquisition time (3.4918 s)	2.4918 s	99.96	0.12
	4.4918 s	100.02	0.06
Spectral width (15 ppm)	10 ppm	99.88	0.20
	20 ppm	99.96	0.12

**Table 6:** Evaluation data of recovery studies using UV–vis spectrophotometer.

Recovery Level (%)	Theoretical Amount Concentration ^1^ (µg/mL)	Amount Added Concentration ^1^ (µg/mL)	%Recovery	%RSD
80	0.65740	0.52590	100.25	1.22711
100	0.65740	0.65740	103.40	1.06936
120	0.65740	0.78890	105.37	1.41745

^1^ Each value is the average of three determinations.

**Table 7:** Statistical validation for repeatability studies using UV-vis spectrophotometer.

Concentration (µg/mL)	Absorbance	Statistical Analysis
20	0.771	Mean = 0.773 SD = 0.004 %RSD = 0.569
20	0.766	
20	0.773	
20	0.775	
20	0.770	
20	0.778	

**Table 8:** Results of intraday precision using UV-vis spectrophotometer.

Concentration Taken (µg/mL)	Absorbance ^1^			Mean	SD	%RSD
	10:30 a.m.	12:30 p.m.	2:30 p.m.			
20	0.769	0.765	0.760	0.765	0.004	0.558
30	1.081	1.074	1.068	1.074	0.007	0.640
40	1.386	1.377	1.366	1.376	0.010	0.718

^1^ Each value is the average of three replicates analysis.

**Table 9:** Results of interday precision using UV-vis spectrophotometer.

Concentration Taken (µg/mL)	Absorbance ^1^			Mean	SD	%RSD
	Day 1	Day 2	Day 3			
20	0.754	0.747	0.733	0.746	0.011	1.446
30	1.059	1.049	1.030	1.046	0.015	1.412
40	1.358	1.348	1.330	1.345	0.014	1.052

^1^ Each value is the average of three replicates analysis.

**Table 10:** Statistical validation for robustness studies using UV-vis spectrophotometer.

Conc. (µg/mL)	339 nm		344 nm		349 nm	
	Absorbance	Statistical Analysis	Absorbance	Statistical Analysis	Absorbance	Statistical Analysis
20	0.746	Mean = 0.746 SD = 0.005 %RSD = 0.608	0.748	Mean = 0.748 SD = 0.004 %RSD = 0.554	0.724	Mean = 0.725 SD = 0.004 %RSD = 0.557
20	0.753		0.755		0.731	
20	0.741		0.744		0.721	
20	0.744		0.747		0.724	
20	0.742		0.744		0.720	
20	0.749		0.750		0.727	

**Table 11:** Statistical validation for ruggedness studies using UV-vis spectrophotometer.

Analyst 1: Dai Chuan Tan			Analyst 2: Alexandra Quek		
Concentration (µg/mL)	Absorbance	Statistical Analysis	Concentration (µg/mL)	Absorbance	Statistical Analysis
20	0.772	Mean = 0.780 SD = 0.008 %RSD = 1.038	20	0.779	Mean = 0.786 SD = 0.008 %RSD = 1.033
20	0.784		20	0.771	
20	0.790		20	0.775	
20	0.789		20	0.781	
20	0.795		20	0.794	
20	0.787		20	0.779	

**Table 12:** Results of extracts using qNMR and UV–vis methods.

Results	qNMR	UV-vis
%Label claim	101.80	103.69
%RSD	1.24	0.82
Scopoletin content		
*Paederia foetida*		
Hexane	ND	2.10% (20.99 mg/g)
Chloroform	7.34% (73.44 mg/g)	3.85% (38.54 mg/g)
Methanol	ND	1.20% (11.99 mg/g)
*Melicope latifolia*		
Hexane	7.31% (73.11 mg/g)	6.75% (67.46 mg/g)
Chloroform	11.75% (117.49 mg/g)	10.67% (100.67 mg/g)
Methanol	5.61% (56.07 mg/g)	4.05% (40.47 mg/g)

ND means not detected because of the absence of peaks in the ^1^H-NMR.
